# Comparison of bailout and planned rotational atherectomy for severe coronary calcified lesions

**DOI:** 10.1186/s12872-020-01645-4

**Published:** 2020-08-15

**Authors:** Cheng-fu Cao, Yu-liang Ma, Qi Li, Jian Liu, Hong Zhao, Ming-yu Lu, Wei-min Wang

**Affiliations:** Department of Cardiology, Peking University People’s Hospital, Beijing, China

**Keywords:** Severe coronary calcified lesions, Planned rotational atherectomy, Bailout rotational atherectomy

## Abstract

**Background:**

To compare outcomes of bailout and planned rotational atherectomy (RA) in the treatment of severe calcified coronary lesions.

**Methods:**

Data of patients treated with RA from 2017 to 2018 at a single-center registry were retrospectively analyzed. All patients were divided into planned RA and bailout RA groups, data between two groups were compared.

**Results:**

A total of 190 patients were included in this study, 138 patients received planned RA and 52 patients received bailout RA. Baseline clinical characteristics had no significant differences between groups. The number of implanted stents and total stents length were similar. But the number of balloon (1.6 ± 0.8 vs. 2.7 ± 1.3, *P* < 0.001), procedure time (83.5 ± 26.2 vs. 100.8 ± 36.4 min, *P* = 0.007), fluoroscopy volume (941 ± 482 vs. 1227 ± 872 mGy, *P* = 0.012] and contrast amount (237 ± 62 vs. 275 ± 90 ml, *P* = 0.003) were all lower in planned RA group. Planned RA had a higher procedural success rate (99.3% vs. 92.3%, *P* = 0.007) and a lower complication incidence (4.3% vs. 17.3%, *P* = 0.009). But the primary outcomes at 3 years (9.2 and 16.6%, log rank *p* = 0.24) had no difference between groups.

**Conclusions:**

For severe coronary artery calcification, although planned RA did not improved the long term prognosis compared with bailout RA, but it can improve the immediate procedural success rate, reduce the incidence of complications, the procedure time and the volume of contrast.

## Background

Calcified coronary lesions remain a challenge for percutaneous coronary intervention (PCI). Calcified lesions can lead to stent implantation failure or incomplete stent expansion, thus affecting the long-term efficacy of stents. It also increases the risk of perforation and coronary dissection during procedure [1, 2]. Rotational atherectomy RA plays a key role in the therapy of severe calcified lesions [3, 4]. Although routine RA does not improve the clinical outcomes in the ROTAXUS trial, it can significantly improve the success rate of procedure [5]. However, it should be noted that more than 50% patients in ROTAXUS study were only moderately calcified lesions shown by coronary angiography, and 12.5% of the patients in the conventional balloon pre-expansion group needed to cross over to RA because of balloon failure of devices. Therefore, it is of great clinical significance to explore when to start RA therapy for severe coronary artery calcification. In the Rotate multicenter registry planned RA appears to be safe and was associated with a reduction in procedural and fluoroscopy times, contrast volume, and the number of pre-dilation balloon catheters used [6].

The aim of this study was to investigate in a single high volume PCI center the safety and procedural feasibility of a planned RA strategy for the treatment of severely calcified coronary lesions in comparison to a bailout RA approach following device failure.

## Methods

### Study design and population

**Fig. 1:**
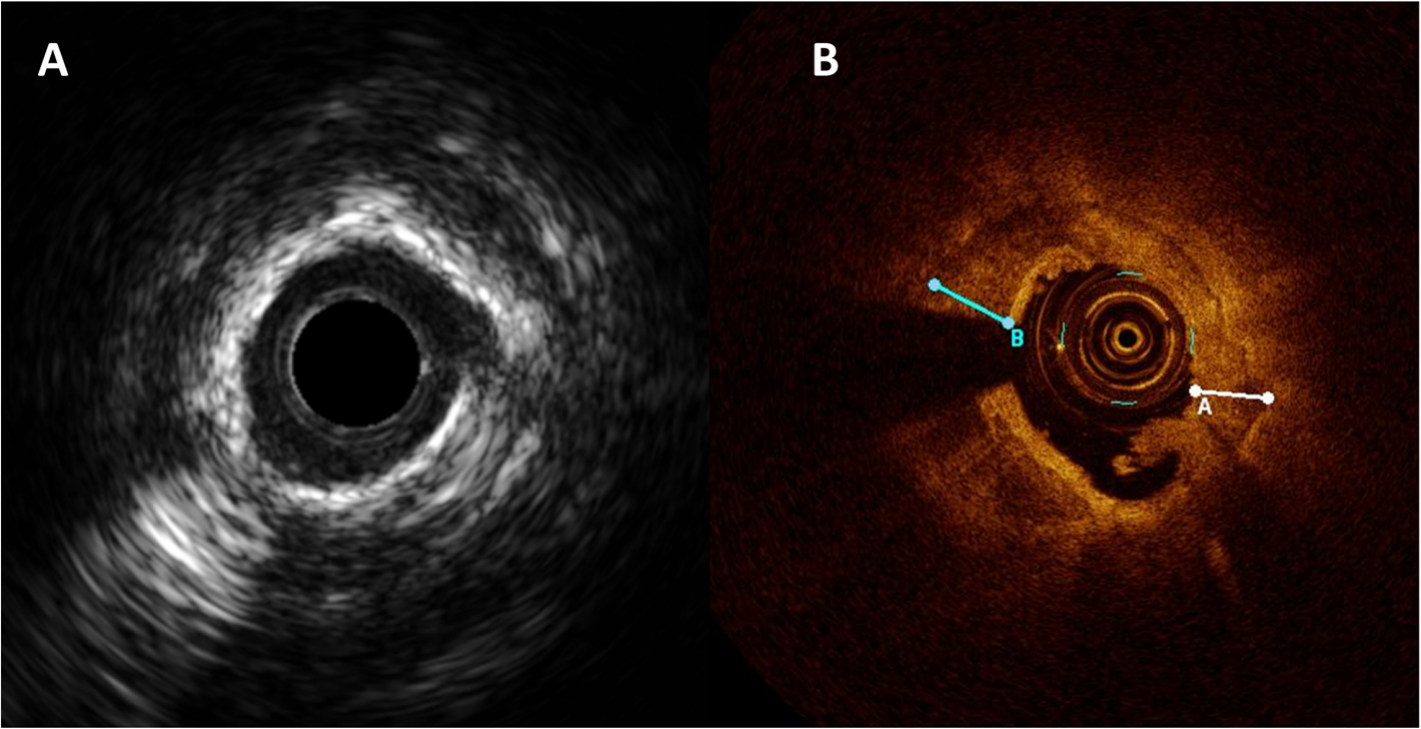
A representative calcification lesion images of IVUS/OCT. **a** Shows 360° calcium with a minimum lumen area 2.1 mm^2^. **b** Shows 360°calcium with maximum calcium thickness 0.58 mm and minimum lumen area1.8 mm^2^

This is a retrospective single-center study of all patients who underwent PCI using RA because of heavily calcified lesions at our hospital from January 2017 to December 2018. Severely calcified lesion was defined as: radiopacities noted without cardiac motion before contrast injection, generally compromising both sides of the arterial lumen. All the patients were divided into 2 groups according to the indication for RA. Planned RA was defined as initial strategy without previous device failure. Bailout RA was defined as RA after incomplete expansion of balloon or failure delivery of any devices. Pre-intervention intravascular ultrasound (IVUS) or optical coherence tomography (OCT) was performed in partial patients, the patients will receive planned RA if the IVUS/OCT showed calcium angle> 270°and calcium length > 5 mm (Fig. 1) or the IVUS/OCT catheter cannot cross the lesions. When discharge from hospital, all the patients were received standard pharmacological treatment (double antiplatelet therapy at least 1 year), and followed-up regularly in clinic.

### Data collection

The demographic and clinical characteristics of all patients included age, gender, medical history, left ventricular function on admission, lipid profiles, serum creatinine, hemoglobin and procedual costs. The angiographic and procedural characteristics included number of diseased vessels, target vessel, the size of burr, total number of stents and total length of stents.

The primary outcome (all-cause death, target vessel revascularization and stent thrombosis) at 3 years were collected during follow-up by telephone or electronic record system.

### Procedural details

Before procedural, all patients received an oral loading dose of 300 mg aspirin and 300 mg clopidogrel. During procedural, all patients received unfractionated heparin at a dose of 70–100 U/kg to maintain an activated clotting time (ACT) > 300 s. In both groups, the choice of vascular access, burr size, IVUS/OCT was left at the operators’ discretion. The IVUS/OCT catheter was advanced beyond the target lesion using a commercially available IVUS/OCT system (40 MHz IVUS catheter; OptiCross, Boston Scientific. ILUMIEN C7-XR, Abbott). RA was performed by using the Rotablator (Boston Scientific Scimed, Inc., Maple Grove, MN, USA). The burr size was selected to reach a burr/vessel ratio of 0.5–0.6. RA speed ranged between 150,000 and 180,000 rotation per minute. Each RA time was 10–15 s. During RA, A continuous intracoronary infusion of a cocktail with unfractionated heparin and nitroglycerin was employed. Success of RA was defined as complete expansion of balloon of target lesion after RA.

### Study endpoints and definitions

Procedural success was defined as a final residual stenosis<30% after stents and grade 3 TIMI flow. Procedural outcomes included total number of balloon and stents used, procedure time, fluoroscopy amount, and volume of contrast used in the two groups. Procedure time was defined as the interval from initial angiography by guiding catheter to final angiography of the target lesion. PCI-related myocardial infarction is defined following the third universal definition of myocardial infarction. Procedure complications included bradycardia, slow-no-reflow, dissection, burr entrapment and perforation. Bradycardia was defined as heart rate less than 45 beats per minute which can influence hemodynamics. Dissection was defined as grades C to F according to National, Heart, Lung, and Blood Institute (NHLBI) criteria [7]. The primary outcome was a composite of all-cause death, target vessel revascularization and stent thrombosis at 3 years.

### Statistical analysis

Statistical analysis was performed using SPSS for Windows 18.0 (SPSS, Chicago, IL). Continuous variables areexpressed as mean ± SD, categorical variables are given as frequencies (%). Univariate comparisons between the two groups were performed using Pearson’s chi-square test for categorical variables, and a Student t test for continuous variables. Kaplan-meier was used to analyze the cumulative incidence of clinical events during the follow-up. Difference was considered to be statistically significant at *P* < 0.05.

## Results

### Patients characteristics

From January 2017 to December 2018, a total of 190 patients were treated with RA. In 138 patients, RA was performed as a planned procedure, while in other 52 patients, RA was as a bailout procedure. In both groups, patients were old, had a higher proportion of hypertension, Diabetes Mellitus (DM), smoking. Most patients presented as stable coronary artery disease and multi-vessel disease. Baseline clinical characteristics had no significant differences between both groups. But the proportion of IVUS/OCT guided PCI was higher in planned RA group than that in bailout RA group (Table 1).

**Table 1:** Baseline Clinical Characteristics of the Study Population

	Planned RA(*n* = 138)	Bailout RA(*n* = 52)	*P* Value
Age (years)	70.2 ± 8.3	69.3 ± 10.1	0.224
Male (%)	86 (62.3%)	36 (69.2%)	0.376
BMI (kg/m^2^)	25.0 ± 3.0	24.7 ± 2.6	0.352
Hypertension (%)	111 (83.3%)	38 (73.1%)	0.272
DM (%)	71 (51.4%)	22 (42.3%)	0.261
Dyslipidemia (%)	35 (25.3%)	18 (34.6%)	0.205
Smoking (%)	60 (43.5%)	26 (50.0%)	0.421
LVEF (%)	65.1 ± 8.2	63.4 ± 9.4	0.289
Prior PCI (%)	30 (21.7%)	12 (23.1%)	0.843
Prior CABG (%)	3 (2.2%)	2 (3.8%)	0.521
eGFR<60 ml/min/1.73m^2^ (%)	16 (11.6%)	9 (17.3%)	0.299
Clinical presentation			0.812
SCAD	31 (22.5%)	11 (21.2%)	
ACS	107 (77.5%)	41 (78.8%)	
MVD (%)	103 (74.6%)	41 (78.8%)	0.740
IVUS/OCT-guided (%)	35 (25.4%)	6 (11.5%)	0.039

*BMI* Body mass index, *DM* Diabetes mellitus, *LVEF* LV ejection fraction, *SCAD* Stable coronary artery disease, *ACS* Acute coronary syndrome, *MVD* Multi-vessel coronary disease

### Lesion and procedural characteristics

The most common RA-target vessel was left anterior descending artery in both groups (58.4 and 59.2% respectively). The most commonly employed burr size was 1.25 mm (41.6%) and 1.5 mm (54.2%). And the use of more than 1 burr was necessary in 5.3% of all cases. The number of stents and the total stents length had no significant differences between both groups, but the number of balloons in bailout RA group was more than that in planned RA group. In addition, the procedure time, fluoroscopy volume and contrast amount were all lower in planned RA group than bailout RA group (Table 2).

**Table 2:** Angiographic and Procedural Characteristics

	Planned RA(n = 138)	Bailout RA(n = 52)	*P* Value
Target vessel			0.031
LM	2	1	
LAD	111	31	
LCX	4	3	
RCA	21	17	
Chronic total occlusion (%)	5 (3.6%)	4 (7.7%)	0.135
ACC/AHA type B2/C (%)	110 (79.7%)	43 (82.7%)	0.569
Predilatation (%)	136 (98.6%)	50 (96.2%)	0.897
Burr size			0.678
1.25	59	20	
1.5	73	30	
1.75	6	2	
More than 1 burr	7 (5.1%)	3 (5.8%)	0.848
Number of balloons	1.6 ± 0.8	2.7 ± 1.3	<0.001
Number of stents	2.0 ± 0.8	2.2 ± 0.8	0.224
Total stents length (mm)	60.7 ± 24.1	62.1 ± 26.0	0.307
Postdilation(%)	137 (99.3%)	50 (96.2%)	0.923
Procedure time (min)	83.5 ± 26.2	100.8 ± 36.4	0.007
Procedure cost (Yuan)	85,090 ± 22,171	93,801 ± 25,923	0.342
Fluoroscopy Volume (mGy)	941 ± 482	1227 ± 872	0.012
Contrast amount (ml)	237 ± 62	275 ± 90	0.003

*LM* Left main, *LAD* Left anterior descending artery, *LCX* Left circumflex artery, *RCA* Right coronary artery

### Procedural complication characteristics

The procedural success was achieved in the majority of cases (97.4%), but was lower in the bailout RA group (92.3% vs. 99.3%, *P* = 0.007). There was no coronary perforation in the two groups. The incidence of complications was higher in bailout RA group (17.3% vs. 4.3%). The most common complications were bradycardia, slow-no-reflow and dissection (Table 3).

**Table 3:** Complications between groups

	Planned RA(n = 138)	Bailout RA(n = 52)	*P* Value
Procedural Success	137 (99.3%)	48 (92.3%)	0.007
PCI-related MI	7 (5.1%)	4 (7.7%)	0.688
Complications	6 (4.3%)	9 (17.3%)	0.009
Bradycardia	2	4	
slow-no-reflow	2	1	
Dissection	2	3	
Burr entrapment	0	1	

### Long time outcomes

**Fig. 2:**
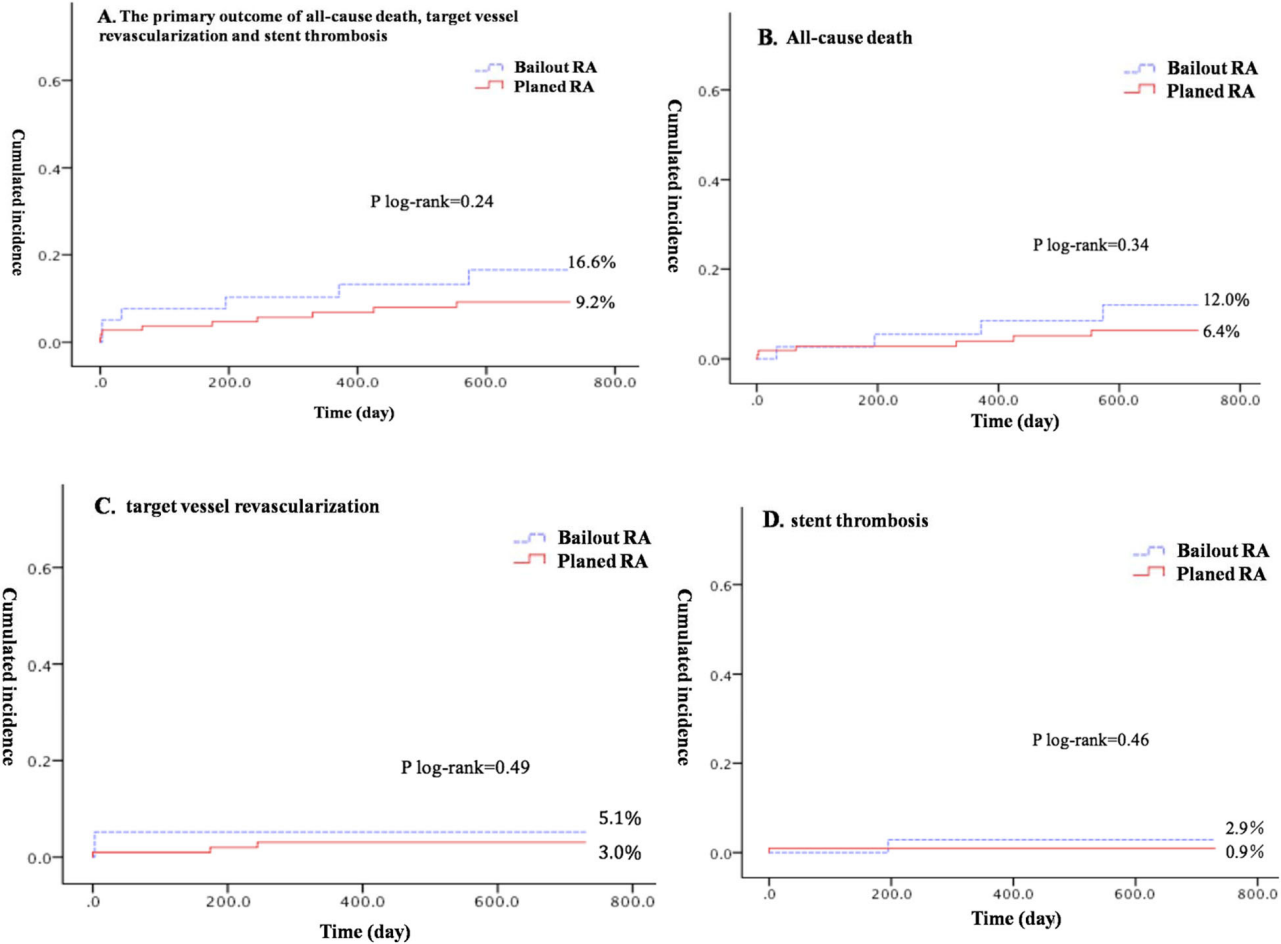
The primary outcomes between groups

All patients were followed up for a long period, with a median follow-up time of 36 months (1–84 months). At 3 years, the primary outcomes (a composite of all-cause death, target vessel revascularization and stent thrombosis) had occurred in 9.2% in the planned RA group and 16.6% in the bailout RA group (log rank p 0.24), and the cumulative 3-year incidences of all-cause death were 6.4 and 12.0% (log rank p 0.34), the 3-year cumulative rates of target vessel revascularization were 3.0 and 5.1% (log rank p 0.49), and the 3-year cumulative rates of stent thrombosis were 0.9 and 2.9% (log rank p 0.46) in planed RA and bailout out RA group respectively. There was no significant difference between groups (*p* > 0.05) (Fig. 2).

## Discussion

The low success rate and high incidence of complications of severe calcification lesions have always been the difficulty during PCI. RA can significantly improve the success rate. Studies showed that the success rate of RA was 95–96.4% [8, 9]. In our study, the success rate of RA was 97.4%. Especially, the success rate in planned RA group was 99.3%, which was significantly higher than that in bailout RA group (92.3%), suggests that planned RA can significantly improve the success rate for severe coronary artery calcification. There were no differences of long term prognosis between groups.

The complications during RA cannot be ignored. Previous studies [10–12] have shown that common complications and the incidence during RA were coronary dissection (10.5%), severe coronary spasm (1.6–6.6%), acute vascular occlusion (3.1%), slow-no-reflow (1.2–7.6%) and coronary artery perforation (0–2%). The most common complications in our study were bradycardia (3.2%), coronary dissection (2.6%), slow-no-reflow (1.6%) and burr entrapment (0.5%). The incidence of complication is high in bailout RA group than that in planned RA group (17.3% vs. 4.3%), which shows that planned RA is more safe than bailout RA.

RA is a complicated and time-consuming procedure in the treatment of severe calcified lesions, which may lead to the increase of procedure cost, contrast volume, procedure time and fluoroscopy amount, and thus increase the economic burden of patients and the risk of contrast-induced nephropathy after procedure. However, there are few studies on this issue. Our study showed that compared with bailout RA, planned RA could significantly reduce the procedure time, the volume of contrast used and the fluoroscopy volume during the procedure, which is important for both the interventional cardiologist and the patients. It is consistent with the results of ALLALI et al. [13]. The ROTATE study showed that compared with bailout RA, planned RA did not reduce the adjusted 1-year MACE [6]. The results of ALLALI showed that although planned RA could improve the success rate of the procedure, but the incidence of 2-years MACE did not decrease compared with bailout RA (25.2% vs 28.7%, log rank *P* = 0.52) [13].

The major limitation of this study is its non randomized design in which operator bias and unmeasured confounders may prohibit definitive conclusions. Limited number of patients, especially in bailout RA group, and not randomized retrospective study design could bias the outcomes.

## Conclusions

Overall, our study showed that, for severe coronary artery calcification, although planned RA did not improve the long term prognosis than bailout RA, but it can improve the immediate procedural success rate, reduce the incidence of complications, the procedure time and the volume of contrast compared with bailout RA.

## Data Availability

The datasets used during the current study are available from the corresponding author on reasonable request.

## Abbreviations

PCI: Percutaneous coronary interventions
RA: Rotational atherectomy
IVUS: Intravascular ultrasound
OCT: Optical coherence tomography
ACT: Activated clotting time
DM: Diabetes mellitus
